# Wavefront-optimized surface retreatments of refractive error following previous laser refractive surgery: a retrospective study

**DOI:** 10.1186/s40662-016-0034-x

**Published:** 2016-02-11

**Authors:** Kevin M. Broderick, Rose K. Sia, Denise S. Ryan, Richard D. Stutzman, Michael J. Mines, Travis C. Frazier, Mark F. Torres, Kraig S. Bower

**Affiliations:** Ophthalmology Service, Walter Reed National Military Medical Center, 8901 Wisconsin Avenue, Bethesda, MD 20814 USA; Warfighter Refractive Eye Surgery Program and Research Center, Ft. Belvoir, VA USA; Ophthalmology Service, Madigan Army Medical Center, Tacoma, WA USA; The Wilmer Eye Institute, Johns Hopkins University, Baltimore, MD USA

**Keywords:** Wavefront-optimized, PRK, LASIK, LASEK, Retreatment, Enhancement

## Abstract

**Background:**

Retreatments are sometimes necessary to correct residual or induced refractive errors following refractive surgery. Many different combinations of primary treatment methods and retreatment techniques have been studied, however, few studies have investigated wavefront-optimized (WFO) technology for retreatment following primary refractive surgery. This study aimed to report the outcomes of WFO photorefractive keratectomy (PRK) retreatments of refractive error following previous laser refractive surgery with PRK, laser in situ keratomileusis (LASIK), or laser-assisted subepithelial keratectomy (LASEK).

**Methods:**

We reviewed records of patients who underwent WFO PRK retreatments using the Allegretto Wave Eye-Q 400 Hz Excimer Laser System (Alcon Surgical) between January 2008 and April 2011 at Walter Reed Army Medical Center and Madigan Army Medical Center. Outcomes were recorded in terms of uncorrected distance visual acuity (UDVA), manifest refraction spherical equivalent (MRSE), corrected distance visual acuity (CDVA), and complications at 1 month (M), 3 M, and 6 M post-op.

**Results:**

Seventy-eight patients (120 eyes) underwent WFO PRK retreatment during the study period. Primary surgery was surface ablation in 87 eyes (78 PRK, 9 LASEK) and LASIK in 33 eyes. The mean spherical equivalent before retreatment was −0.79 ± 0.94 D (−3.00 to 1.88 D). UDVA was ≥ 20/20 in 69 eyes (60.0 %) at 1 M, 54 eyes (71.1 %) at 3 M, and 27 eyes (73.0 %) at 6 M follow-up. MRSE was within ±0.50 D of emmetropia in 78 eyes (67.8 %) at 1 M, 59 eyes (77.6 %) at 3 M, and 25 eyes (67.6 %) at 6 M follow-up. CDVA was maintained within ±1 line of pre-op in 113 of 115 eyes (98.3 %) at 1 M, 74 of 76 eyes (97.4 %) at 3 M, and 37 eyes (100 %) at 6 M follow-up.

**Conclusion:**

Although follow-up was limited beyond 3 M, WFO PRK retreatments in patients with residual refractive error may be a safe and effective procedure. Further studies are necessary to determine the long-term safety and stability of outcomes.

## Background

Laser refractive surgery is one of the most commonly performed eye surgeries worldwide and has been established to be successful in correcting refractive errors. With conventional refractive surgery, a patient may not achieve his or her maximal visual quality potential post-operatively as a result of changes in corneal shape and subsequent induction of ocular aberrations [1]. Several studies have shown that conventional methods result in the induction of higher order aberrations, including spherical aberration and coma, which can lead to negative visual outcomes [2, 3]. The advent of wavefront technology has greatly improved our understanding of ocular aberration, and with its incorporation into refractive surgery techniques, patients are experiencing better visual outcomes and greater satisfaction when compared with conventional methods [1, 4, 5]. Even with these improved outcomes, however, retreatments are sometimes necessary to correct residual or induced refractive error following refractive surgery.

Retreatment following refractive surgery is a subject that has been extensively studied and has been observed in many different combinations of primary treatment methods and retreatment methods. The overall rate for retreatment varies widely in the literature with the average rate ranging from 5.5 % to 8.3 % for primary myopic laser in situ keratomileusis (LASIK) [5]. Prior to the advent of wavefront technology, flap-lift retreatments following conventional LASIK was shown to be safe and effective in multiple studies [6–9]. Jin and Merkley [10] illustrated that retreatment using conventional LASIK is safe, effective, and predictable following wavefront-guided (WFG) and standard myopic LASIK. They also compared conventional to WFG retreatments following primary conventional LASIK treatments in another study [11]. Kanellopoulous and Lawrence [12], along with several other groups, illustrated similar findings following WFG retreatments of primary conventional LASIK. Kashani et al. [5] observed that WFG retreatments following primary WFG surgeries in both myopes and hyperopes had favorable outcomes with respect to safety, predictability, and efficacy. Few studies, however, have reported outcomes specifically of wavefront-optimized (WFO) retreatments following refractive surgery.

To our knowledge, the only recent study addressing retreatment data using WFO technology was a study by Randleman et al. [13], which looked primarily at retreatment rates after WFO ablation and examined potential risk factors for retreatment, including age, sex, corneal characteristics, and environmental factors. All patients involved in that study underwent either WFO photorefractive keratectomy (PRK) or LASIK for both their primary and retreatment surgeries. In this study, we report the visual outcomes of WFO PRK retreatment following PRK, LASIK, and laser-assisted subepithelial keratectomy (LASEK).

## Methods

We performed a retrospective database review of patients who underwent WFO PRK retreatments for residual refractive error following previous laser refractive surgery at either the Walter Reed Army Medical Center (WRAMC) Center for Refractive Surgery (CRS) or the Madigan Army Medical Center (MAMC) Refractive Surgery Center (RSC) between January 2008 and April 2011. As part of the U.S. Army Warfighter Refractive Eye Surgery Program (WRESP), each refractive center maintains separate outcomes databases as computerized spreadsheets (Microsoft Excel, Redmond, Washington) to track patient outcomes and follow-up. In addition to patient demographic information, these databases list pre-operative refractive error, primary treatment performed, type of retreatment performed, if any, outcomes, and any complications. Data from patients identified as having undergone WFO PRK retreatments were extracted from these databases and compiled in a new computerized spreadsheet for analysis. Prior to review of these databases, approval for the study was obtained from the institutional review board (Department of Clinical Investigation) at both WRAMC and MAMC.

All patients were active duty personnel participating in the WRESP who gave informed consent to undergo the retreatment procedure for residual refractive error. WRESP patients are typically 20 to 50 years old and predominantly (74.4 %) male. All active duty soldiers with at least 18 months of service obligation remaining at the time of surgery are potentially eligible for participation in the program, with preference given to combat arms soldiers [14]. Pre-operative evaluation of refractive candidates included uncorrected distance visual acuity (UDVA), corrected distance visual acuity (CDVA), manifest and cycloplegic refractions, keratometry and ultrasound corneal pachymetry, contact tonometry, and comprehensive ophthalmic exam. Patients with active ophthalmic disease, keratoconus, glaucoma, ocular herpes simplex or herpes zoster, significant corneal neovascularization, clinically significant lens opacity, medical conditions that may impair healing (e.g. collagen vascular disease, autoimmune disease, immunodeficiency disease), lack of refractive stability within 6 months prior to surgery, suspicious corneal topography, and pregnancy were excluded from treatment. There were no set criteria in determining the need for a retreatment procedure; this was generally decided by the patient and physician.

Overall, reasons for residual refractive error following initial laser refractive treatment include initial undertreatment, overtreatment, or regression. During evaluation for retreatment, each patient obtained corneal tomography to assess for and rule out evidence of ectasia or forme-fruste keratoconus. If concerning corneal tomography was present, the patient was no longer considered a candidate for refractive surgery. No patients included in the study showed evidence (either pre or post-operatively) of corneal ectasia during the study period.

All retreatments were WFO ablations with Allegretto Wave Eye-Q 400 Hz Excimer Laser System (Alcon Surgical, Fort Worth, Texas). The epithelium was debrided mechanically using either a 9.0 mm Amoils rotating scrubber brush (Innovative Excimer Solutions, Toronto, Canada) or with a dilute solution of 20 % alcohol for approximately 30 s, depending on surgeon preference. Prophylactic mitomycin-C (MMC) was used in 69 (57.5 %) retreatment cases, at either 0.01 % or 0.02 % concentration, and was applied to the stromal bed immediately following ablation for times ranging between 15 and 60 s, depending on surgeon preference.

The post-operative management of patients varied little between surgeons and generally consisted of the following treatment plan: topical moxifloxacin hydrochloride 0.5 % ophthalmic solution (Vigamox, Alcon Laboratories, Fort Worth, Texas) four times daily for one week; topical prednisolone acetate 1 % ophthalmic solution (Pred Forte, Allergan Inc., Irvine, California) every two hours for the first three days followed by four times daily for the remainder of the first week followed by a six-week taper; topical ketorolac tromethamine 0.4 % ophthalmic solution (Acular-LS, Allergan Inc., Irvine, California) up to four times daily during the first 48 h as needed for pain; and frequent lubrication with preservative-free artificial tears. A high oxygen transmissible soft contact lens was placed on all eyes at the time of surgery and removed after complete re-epithelialization, typically between four and seven days. Postoperative data from regularly scheduled follow-up visits at one, three, and six months included UDVA, manifest refraction, manifest refraction spherical equivalent (MRSE), CDVA and complications, including but not limited to corneal haze, dry eyes, and steroid response ocular hypertension or glaucoma.

Visual outcomes were documented in terms of standard measures of efficacy, safety predictability and stability. Efficacy was measured as the number (%) of eyes achieving UDVA 20/20. Safety was measured in terms of maintenance of CDVA within one line of preoperative. Loss of more than one line CDVA from any cause was considered a complication. Predictability was measured as the number (%) of eyes with MRSE within ±0.50 diopter (D) of emmetropia. Refractive stability was measured as the number (%) of eyes with < 0.50 D change in MRSE over a minimum six month follow-up period. Safety index was calculated as the ratio of mean postoperative CDVA over mean preoperative CDVA. Efficacy index was measured by ratio of mean postoperative UDVA over mean preoperative CDVA. Visual outcomes were also graphically presented using a standard format [15].

## Results

Between January 2008 and April 2011, 78 patients (120 eyes) underwent WFO PRK retreatment for residual refractive error at either WRAMC or MAMC. Table 1 summarizes the preoperative clinical and demographic characteristics of these patients. The primary surgery was surface ablation in 87 eyes (78 PRK, 9 LASEK) and LASIK in 33 eyes. MMC was used in 69 (57.5 %) of the retreatment cases at either 0.01 % or 0.02 % concentration for time periods ranging between 15 and 60 s. Surface epithelium was debrided by the Amoils rotating scrubber brush in 22 eyes and 20 % alcohol in 98 eyes. There were no intraoperative complications. Follow-up rate was 115 out of 120 eyes (95.8 %) at one month, 76 eyes (63.3 %) at three months, and 37 eyes (30.8 %) at six months.

**Table 1:** Preoperative characteristics of patients who underwent WFO PRK retreatments following previous laser refractive surgery with PRK, LASEK, or LASIK between January 2008 and April 2011 at Walter Reed Army Medical Center or Madigan Army Medical Center

	78 patients (120 eyes)
Age ± SD (years)	40.3 ± 8.3 (26–58)
Gender (% Female)	20 (25.6 %)
Sphere ± SD (D)	−0.46 ± 1.08 (−3.00 to 2.00)
Cylinder ± SD (D)	−0.67 ± 0.64 (0 to −2.50)
MRSE ± SD (D)	−0.79 ± 0.94 (−3.00 to 1.88)
UDVA ± SD (logMAR)	0.31 ± 0.17 (0 to 1.00)
Snellen equivalent (20/x)	40 (20 to 200)
CDVA >20/20 (% of eyes)	100 %
Average Keratometry ± SD (D)	41.33 ± 1.91 (36.85 to 46.05)
Central Corneal Thickness ± SD (μm)	493 ± 46 (392 to 623)
Primary surgery (% surface ablation)	57 (73.1 %)
Epithelial Removal Technique (20 % ethanol)	81.7 %

*MRSE=* manifest refraction spherical equivalent, *UDVA=* uncorrected distance visual acuity, *CDVA=* corrected distance visual acuity

**Fig. 1:**
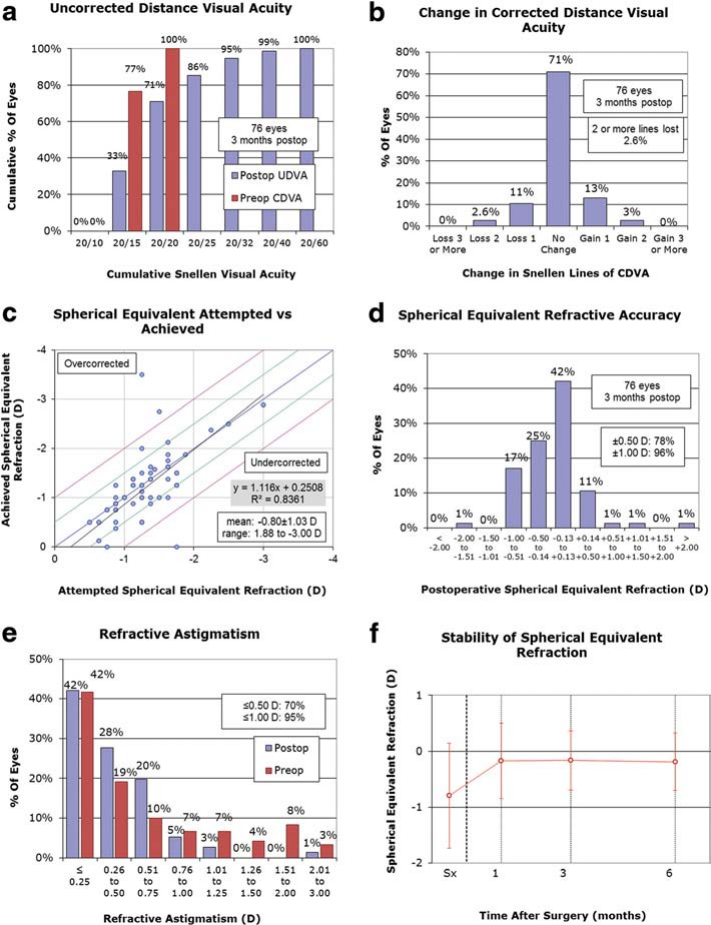
Three-month outcomes of wavefront-optimized PRK retreatment. **a** Uncorrected distance visual acuity. **b** Change in corrected distance visual acuity. **c** Spherical equivalent attempted vs. achieved. **d** Spherical equivalent refractive accuracy. **e** Refractive astigmatism **f** Stability of spherical equivalent refraction

UDVA was ≥ 20/20 in 69 eyes (60.0 %) at one month, 54 eyes (71.1 %) at three months, and 27 eyes (73.0 %) at six months follow-up. CDVA was maintained within ±1 line of pre-op in 113 of 115 eyes (98.3 %) at one month, 74 of 76 eyes (97.4 %) at three months, and 37 eyes (100 %) at six months follow-up. MRSE was within ±0.50 D of emmetropia in 78 of 115 eyes (67.8 %) at one month, 59 of 76 eyes (77.6 %) at three months, and 25 of 37 eyes (67.6 %) at six months follow-up. Based on available data at one and six months postoperatively, MRSE changed < 0.5 D between one and six month follow-up in 21 of 35 eyes (60.0 %). The three month postoperative visual outcomes are shown in Fig. 1. Of the 76 eyes followed at three months postoperatively, 59 eyes (77.6 %) had UDVA within one line of their preoperative CDVA. Safety and efficacy indices progressively improved over six months postoperatively (Table 2). Dry eye was the most commonly reported post-operative complication throughout the follow-up period: 16 eyes (13.9 %) at one month, 16 eyes (21.1 %) at three months, and 2 eyes (5.4 %) at six months. No clinically significant corneal haze developed in any of the eyes post-operatively. Corneal haze of grade 1+ or less was noted in 13 eyes (11.3 %) at one month and 6 eyes (7.9 %) at three months. All cases of corneal haze resolved without surgical intervention by six months. One patient (2 eyes) had loss of two lines of CDVA at one month follow-up, but subsequently regained them at three month follow-up without intervention. A separate patient (2 eyes) had no complications at one month follow-up but had loss of two lines of CDVA at three months, and subsequently was lost to further follow-up. Table 3 summaries all complications over the six month follow-up period.

**Table 2:** Safety and efficacy indices of wavefront-optimized PRK retreatment

Follow up (month)	Safety Index	Efficacy Index
1	0.91	0.73
3	1.00	0.81
6	1.03	0.84

Safety index (post-op CDVA / pre-op CDVA)

Efficacy index (post-op UDVA / pre-op CDVA)

**Table 3:** Post-operative complications

Post-op complications	1 M	(*n* = 115)	3 M	(*n* = 76)	6 M	(*n* = 37)
	No.	%	No.	%	No.	%
None	79	68.7	53	69.7	35	94.6
Dry eye	16	13.9	16	21.1	2	5.4
Corneal haze (trace)	10	8.7	4	5.3	0	0.0
Corneal haze (1+)	3	2.6	2	2.6	0	0.0
Corneal haze (2+ or >)	0	0.0	0	0.0	0	0.0
Corneal scar	0	0.0	0	0.0	0	0.0
Residual astigmatism	6	5.2	2	2.6	0	0.0
Induced astigmatism	6	5.2	1	1.3	0	0.0
Undercorrected	3	2.6	3	3.9	2	5.4
Overcorrected	11	9.6	1	1.3	1	2.7
Regression	0	0.0	0	0.0	0	0.0
Corneal abrasion	0	0.0	0	0.0	0	0.0
Delayed epithelial healing	0	0.0	0	0.0	0	0.0
Recurrent corneal erosion	0	0.0	0	0.0	0	0.0
Steroid responder	0	0.0	0	0.0	0	0.0
Corneal infiltrate	0	0.0	0	0.0	0	0.0
Other	0	0.0	0	0.0	0	0.0

### Primary LASIK

Thirty-three eyes had LASIK as primary treatment. The mean spherical equivalent before retreatment was −0.70 ± 0.86 D (−1.75 to 1.25 D). UDVA was ≥ 20/20 in 12 of 31 eyes (38.7 %) at one month, 18 of 24 eyes (75.0 %) at three months, and 5 of 6 eyes (83.3 %) at six months follow-up. CDVA was maintained within ±1 line of pre-op in all eyes at one month (31 eyes), three months (24 eyes), and six months follow-up (6 eyes). MRSE was within ±0.50 D of emmetropia in 17 of 31 eyes (54.8 %) at one month, 19 of 24 eyes (79.2 %) at three months, and 4 of 6 eyes (66.7 %) at six months follow-up. Based on available data at one and six months postoperatively, MRSE changed < 0.5 D between one and six month follow-up in 2 of 6 eyes (33.3 %).

### Primary surface ablation

Eighty-seven eyes had surface ablation (78 PRK, 9 LASEK) as primary treatment. The mean spherical equivalent before retreatment was −0.83 ± 0.97 D (−3.00 to 1.88 D). UDVA was ≥ 20/20 in 57 of 84 eyes (67.9 %) at one month, 36 of 52 eyes (69.2 %) at three months, and 22 of 31 eyes (71.0 %) at six months follow-up. CDVA was maintained within ±1 line of pre-op in 82 of 84 eyes (97.6 %) at one month, 50 of 52 eyes (96.2 %) at three months, and 31 eyes (100 %) at six months follow-up. MRSE was within ±0.50 D of emmetropia in 61 of 84 eyes (72.6 %) at one month, 40 of 52 eyes (76.9 %) at three months, and 21 of 31 eyes (67.7 %) at six months follow-up. Based on available data at one and six months postoperatively, MRSE changed < 0.5 D between one and six month follow-up in 19 of 29 eyes (65.5 %).

## Discussion

Wavefront technology has greatly improved our understanding of ocular aberration, and with its incorporation into refractive surgery techniques, patients are experiencing better visual outcomes and greater satisfaction when compared to conventional methods [2, 5]. In multiple studies, both WFG and WFO refractive surgeries have been found to achieve essentially similar visual outcomes [2, 4, 16]. The use of WFG technology for refractive enhancement has also been shown to be safe and effective [5]. However, the visual outcomes following WFO retreatment have not yet been fully established.

From our review, retreatment using WFO PRK retreatment following primary refractive surgery with PRK, LASEK, or LASIK appears to be a safe, effective, and predictable treatment method for correcting residual or induced refractive error. Though our reduced long-term follow-up rate limits our ability to comment on the long-term stability of retreatment cases, our results indicate that retreatments appear stable through to the six month post-operative period. The results from our study are consistent with results from the WFO retreatment study by Randleman et al., although their study focused primarily on the rate of retreatment and the factors influencing that rate, as opposed to visual outcomes from the retreatment procedure [13]. Their study also briefly noted WFO retreatment outcomes (UDVA), but did not comment on the safety of this platform as a retreatment method or on any post-retreatment complications [13]. In our study, there were no major adverse events following WFO PRK retreatments. Dry eye was the most common complication seen in this study, but we were unable to determine if the dry eye symptoms were worse prior to retreatment or if these symptoms became chronic given the limited follow-up duration. Our study was limited primarily by low patient follow-up after three months. This was most likely due to patients’ various active duty requirements, such as training exercises and deployments, which precluded patients from attending follow-up appointments. Ideally, more of our patient population would return for regular re-visits and for a longer follow-up duration to better assess refractive outcomes; however, with all of our subjects being active duty military personnel, a large portion of our patients were moved to other military facilities, sent for prolonged training assignments, or deployed to Afghanistan or Iraq for 12–15 month-long tours, during the follow-up period. As a result, our ability to comment on the refractive stability beyond the 6 month period is limited. Further limitations to our study include surgical variability between individual retreatment cases. Surgical variables that varied largely based on surgeon preference included the method of epithelial debridement (brush or alcohol), the decision to use MMC, and if used, the concentration and exposure time of MMC. The actual effect of these surgical variables on visual outcomes has widely been debated. The concentration of MMC used, the stromal exposure time to MMC, and the correct situation to use MMC varies considerably between practices and surgeons. A true consensus on the optimal use of MMC had not yet been established in either primary or retreatment cases.

The visual and refractive outcomes from this study are comparable to the outcomes from other retreatment studies. Jin and Merkley [10] compared visual outcomes of conventional LASIK retreatments following either primary WFG LASIK or primary conventional LASIK: 75.0 % of eyes in both of their study groups had UDVA > 20/20 following retreatment. In their WFG group, 91.0 % of eyes were within ±0.50 D of emmetropia, and of their standard primary group, 87.0 % of eyes were within ±0.50 D of emmetropia. In addition, they reported that no eyes lost ≥ 2 lines of CDVA, which is also true in our LASIK population subset. Within our study, 3.3 % of eyes (4 eyes of two patients) lost 2 lines of CDVA; both patients were from our PRK subset group. One of these patients (two eyes) improved and regained CDVA at the next month of follow-up, and the second patient maintained ±1 line CDVA until the last month of follow-up. Our rate of 2.6 % is actually lower than that reported in another Jin and Merkley [11] study where outcomes of conventional and WFG myopic LASIK retreatment were assessed. In their study, they reported that 17 % of eyes in their WFG retreatment group lost 2 lines of CDVA.

Many studies to date have shown very similar and often excellent refractive outcomes following both primary and retreatment cases utilizing either WFG or WFO platforms. [1, 3, 5, 10, 16] Additionally, many studies have shown that following WFG or WFO primary treatments, there is a similar increase in the amount of induced higher order aberrations [1, 17]. Given similar refractive outcomes and induction of higher order aberrations between the two platforms, albeit following primary refractive procedures, our experience was that the WFO platform was best utilized for patients with higher refractive errors or with a significant amount of astigmatism given the faster laser ablation and more peripheral treatment profile. Most retreatment cases, however, will not have markedly high amounts of residual refractive error or astigmatism, so either platform could be utilized by the surgeon.

During the study period, we performed 110 enhancement procedures at WRAMC, which comprised 1.8 % of the total number of refractive surgeries performed at this center during the study period. Though our study did not primarily focus on establishing a retreatment rate during the study period, our experience with retreatment cases appeared to be similar to others cited in the literature. Randleman et al. reported an overall retreatment rate of 6.3 % following WFO PRK and LASIK. Kashani et al. reported a retreatment rate of 3.1 % in their WFG LASIK retreatment study; Netto and Wilson reported a retreatment rate of 14 % in their standard LASIK retreatment study.

## Conclusion

While our study only provides a descriptive narrative of WFO PRK retreatment outcomes, it demonstrates potential outcomes and variables to be investigated in future prospective studies. Our study also tentatively establishes the safety of WFO PRK retreatments following any primary refractive surgery. Since the safety of WFG retreatments has been established in several studies, and WFO retreatment outcomes appear to match those of WFG retreatments, our study allows clinicians to have treatment options when faced with patients needing retreatment. Our study also lays the groundwork for a randomized controlled trial comparing the two platforms in retreatment cases.
